# Customized digestion protocols for copepods, euphausiids, chaetognaths and fish larvae facilitate the isolation of ingested microplastics

**DOI:** 10.1038/s41598-024-70366-x

**Published:** 2024-08-28

**Authors:** Imke Podbielski, Thea Hamm, Mark Lenz

**Affiliations:** 1 GEOMAR Helmholtz Centre for Ocean Research Kiel, Kiel, Germany; 2 The Lower Saxon Wadden Sea National Park Authority, Wilhelmshaven, Germany

**Keywords:** Biological techniques, Ecology, Marine biology, Environmental health, Ecophysiology, Ecosystem ecology, Environmental monitoring, Environmental impact

## Abstract

Degradation of oceanic plastic waste leads to the formation of microplastics that are ingested by a wide range of animals. Yet, the amounts that are taken up, especially by small zooplankton, are largely unknown. This is mostly due to the complex methodology that is required for isolating ingested microplastics from organisms. We developed customised, effective and benign digestion protocols for four important zooplankton taxa (copepods, euphausiids, chaetognaths and fish larvae), and assessed their digestion efficacy and their potential to cause particle loss or to alter microplastics using six polymers (HDPE, LDPE, PS, PET, PVC, PMMA). All protocols are based on an incubation of the organic matrix with 10% KOH at 38 °C, which is optionally combined with digestive enzymes (chitinase, proteinase K). This yielded digestion efficacies of > 98.2%, recovery rates of > 91.8%, < 2.4% change in microplastics’ size, while no visual alteration of the microplastics and no changes in their spectra were observed when analysing them with a hyperspectral imaging camera. The proposed protocols are inexpensive (< 2.15 € per sample), but require several days when enzymatic digestion is included. They will facilitate research on microplastic ingestion by small marine organisms and thus enable well-founded conclusions about the threat that microplastics pose to these animals as well as about the role of biota in determining the vertical distribution of microplastics in oceanic environments.

## Introduction

Marine microplastics (MP) mostly originate from the fragmentation of larger plastic waste at sea^1^ or, to a smaller extent, from the loss of preproduction pellets during transport and the import of car tyre particles from land masses^2^. They can vary considerably in size, chemical composition, shape and abundance. In pollution hotspots such as the Atlantic Garbage Patch, MP can occur in abundances of up to 2154 particles per m^3^ in surface waters^3^.

Zooplankton makes up a significant portion of animal biomass in the world’s oceans^4,5^ and plays a central role in marine food webs^6,7,9^. Within the oceanic garbage patches, zooplankton organisms are permanently exposed to high concentrations of MP^9,10^, but also in other sea areas they may encounter and ingest the micro-sized particles. It is therefore crucial to determine the nature and the amount of MP that is ingested by zooplankton to assess the risks it poses to these animals and to investigate the role that zooplankton may play for the vertical transport of MP in oceanic systems.

According to a recent definition^11^, MP are particles in the size range between 1 and 1000 µm. They are ingested by many marine organisms^12–14^, and this can potentially impair their health and survival^15–17^. MP ingested by zooplankton must be small, since most of these organisms cannot take up large items due to their inflexible mouth parts. Copepods mainly ingest prey items from 11 to 87 µm^18^, chaetognaths from 100 to 500 µm^19^, and euphausiids from 6 to 50 µm^20^. However, experimental laboratory studies already demonstrated that copepods ingest MP in the size range between 2 and 30 µm^15,21^. Small MP are believed to be more detrimental to life forms than large ones, because they are ingested more easily and because toxicity increases with the surface/volume ratio^22,23^. This is putting zooplankton at a particular risk to suffer from this form of marine pollution.

Yet, the majority of studies on the ingestion of MP in the marine environment focussed on fish guts and mussel soft bodies. Although, both groups of animals are important, they are not representative for the majority of marine life forms. This is because most fish species ingest rather large MP, and the surveyed mussel species are restricted to coastal regions^14,24–26^. In the review of Marmara et al.^32^ only 1.8% of the 822 marine species that were observed to ingest MP were zooplankton. Therefore, the distribution of MP in oceanic organisms and their potential impact on them remains largely unknown.

While the abundance of large MP (> 300 µm) has been extensively researched in non-living environments such as beach sediments, water bodies or terrestrial soils (e.g.^27–31^), as well as in large animals such as fish and birds^32^, reports on MP in small-sized animals, which cannot be dissected, are still scarce. This is partially due to the complicated and time-consuming methodology that is required for extracting MP from matrices such as gut systems or tissues. Furthermore, the small size of the ingested MP is aggravating their detection and identification, which requires small filter pore-sizes (~ 10 µm) and analytical devices with a sufficient resolution. In large animals, such as fish and mussels, the prior extraction of the gut system or the separation of the soft body from the shell allows the digestion of body parts that do not contain hard structures^25,33–35^. However, since the small zooplankton organisms cannot be dissected, they have to be digested as a whole, including the robust exoskeletons that contain chitinous or calcareous materials. Incomplete digestions of hard structures, however, may later impede the identification of MP that are contained in the sample.

In recent years, the number of studies that focused on digestion methods to investigate MP concentrations in invertebrates increased substantially^36–38^. Authors compared different reagents with regard to their effects on various polymer types as well as with regard to their digestion efficacy. However, in most cases we lack information about how strongly the digestion efficacy of a given reagent or a combination of reagents varies between organisms and tissue types. For the reasons given above, there is certainly not a “one size fits all” solution for extracting MP from animals. This should be particularly true for the very diverse marine zooplankton that varies largely in anatomy and morphology. It is even likely that many taxa require a customised digestion protocol, and these are not available as of today.

In this study, we focus on customizing digestion protocols for four relevant zooplanktonic taxa: fish larvae, chaetognaths, copepods and euphausiids. They belong to the groups with the highest contributions to total mesozooplankton biomass and abundance in the oceans^5,39^. These customized protocols should enable filtration through small (10 µm) pores and facilitate MP particle identification by spectroscopy. Furthermore, they should aim at minimizing the number of organic fragments in the residue, since most MP analyses still rely on the manual picking of particles prior to the spectroscopic analysis. The presence of non-digested fragments, however, increases the amount of time needed for this work step.

## Results and discussion

Digestion protocols for MP research should be minimal and fast, because every work step (e.g. drying, grinding, washing, handling) increases the risk of particle loss or contaminations. Furthermore, the reagent(s) used as well as incubation temperature should not alter the MP, while the digestion should be as complete as possible, since remaining organic materials aggravate the picking of particles. We tested a wide variety of established, adapted and newly developed digestion procedures with fish larvae, chaetognaths, copepods and euphausiids^12,40^ until optimal results with regard to the mentioned criteria were achieved (Table S2).

In a literature research on existing digestion protocols, we considered 172 studies of which 50 investigated the ingestion of MP by organisms using chemical digestion of dissected tissues or whole animals (Table 1). They employed various digestion reagents: acids (e.g. HNO_3_, HCl), bases (e.g. NaOH, KOH), oxidizing agents (e.g. H_2_O_2_) and enzymes (e.g. proteinase K, trypsin). Most protocols used HNO_3_ or other acids (n = 29), followed by KOH (n = 22) and H_2_O_2_ (n = 17), while using only enzymes was rare (n = 13) (Table S1). Digestion durations ranged from 5 min to 3 weeks and incubation temperatures from room temperature to 100 °C. However, most protocols incubated samples between 24 and 72 h and at a temperature of 60 °C, although temperatures > 40 °C were repeatedly reported to damage MP^33,41,42^. In 62% of the studies, the authors did not test for digestion efficacies and did not assess the potential effects of the applied methodology on MP, while the majority of studies focussed on fish stomachs (n = 21) as well as mussels (n = 23) and to a considerably lesser degree on copepods (n = 2) and euphausiids (n = 1). No protocol was found for chaetognaths (Table 1). Hence, there is a lack of digestion protocols for zooplankton organisms, which can likely be attributed to the fact that the various zooplankton groups and species differ in tissue and exoskeleton characteristics and/or in the exoskeleton/tissue ratio. This makes them candidates for customised approaches.

**Table 1:** Exemplary selection of studies that report digestion protocols using a variety of chemical reagents and that indicate their digestion efficacy, limitations and, partly, potential alteration of MP. A complete overview of all 50 evaluated research articles is available in the SI (Table S1).

Reagent	Step	Temp	Duration	Organism	Tissue	Digestion efficacy	Limitations	MP Types tested	MP Alteration	Source
HNO_3_ (69%)		40 °C, 50 °C, 60 °C	24–48 h	Lobster	Gastrointestinal tract	110%		PP, PS, PET, PA, HDPE, LDPE	severe (PA, PS), intermediate (PP, PET, HDPE, LDPE)	Hara et al.^42^
HNO_3_ (9, 18, 35, 50%)		60 °C	1 h	Bivalve	Whole organism, soft tissue	100%	Tissue residues; minimum amount needed 35%	NY, PET, HDPE, PVC	severe (PET, HDPE, NY)	Catarino et al.^33^
HCl (1 M/2 M)		20˚C	24 h	Zooplankton, Copepods	Whole organism	82%	Digestion incomplete	NA	not tested	Cole et al.^12^
HCl (37%)		25 °C, 40 °C, 50 °C, 60 °C	4 d	Fish	Muscle & skin	99–100%		HDPE, LDPE, PP, PVC, PET, PS, NY6 or NY66	severe (NY6, NY66, PET, PVC)	Karami et al.^25^
H_2_O_2_ (30%)		40 °C	2 d/4 d	Prawn	gastrointestinal tract	94–96%	< 4 d digestion incomplete	PA, PE, PES, PP, PS, PVC, Rayon	4 d: severe (Rayon)	Li et al.^50^
H_2_O_2_ (35%)		25 °C, 40 °C, 50 °C, 60 °C	4 d	Fish	Muscle and skin	87–100%	< 50 °C digestion incomplete; 50 °C foaming	HDPE, LDPE, PP, PVC, PET, PS, NY6 or NY69	< 60 °C: not tested; 60 °C: severe (NY6, NY66, PVC), intermediate (PET)	Karami et al.^25^
NaOH (1 M, 10 M)		60˚C	24 h	Zooplankton, Copepods	Whole organism	Not reported, 91%		PS, Nylon, PE, uPVC, PES	severe (Nylon, PE, PES), intermediate (uPVC)	Cole et al.^12^
NaOH (5 M)		25 °C, 40 °C, 50 °C, 60 °C	4 d	Fish	Muscle and skin	29–91%	Digestion incomplete	NA	Not tested	Karami et al.^25^
KOH (10%)		60˚C	24 h	Fish,Bivalve, Crab	Fillet, soft tissue	Not tested; not tested; 100%		LDPE, HDPE, PP, PA, PS	Severe (CA), intermediate (PET)	Dehaut et al. (2017)
KOH (10%)		25 °C, 40 °C, 50 °C, 60 °C	4 d	Fish	Muscle & skin	97–99%	Most effective protocol 40 °C (2–3 d)	HDPE, LDPE, PP, PVC, PET, PS, NY6 or NY68	> 50 °C: intermediate (NY66); severe (PET, PVC)	Karami et al.^25^
KOH (10%)		60 °C, 40 °C	48 h	Bivalve	Whole organism	91–98%		PS, PP, PET, PA, Acrylic, Rayon, PVC, LDPE	> 40 °C: severe (Rayon)	Thiele et al.^37^
Proteinase K	1	50˚C	2 h	Zooplankton, Copepods	Whole organism	97%	High cost	PS, NY, PE, uPVC, PES	None	Cole et al.^12^
NaClO_4_ (5 M)	2	20 °C	> 20 min							
NaClO_4_ (5 M)	3	60 °C	20 min							
Protease K	1	40 °C	48 h	Prawn	gastrointestinal tract	94–95%	Digestion incomplete	NA	Not tested	Li et al.^50^
Chitinase	2	37 °C	72 h							

"Overnight" is reported as 12 h, Room temperature as 20 °C. Chemical effect on MPs is defined as follows:none—no effects; intermediate—effects on MP types include: coloration, deformation, agglomeration, change in shape; severe—effects on MP types include: dissolution, fragmentation, change in size.

MPs: CA: cellulose acetate; NY: Nylon; PA: polyamide; PE: polyethylene; HDPE: high density PE; LDPE: low density PE; PES—polyester; PET: polyethylene terephthalate; PP: polypropylene; PS: polystyrene; PVC: polyvinyl chloride, uPVC: unplasticized PVC.

Although, previous studies reported good results for acidic digestions and ICES also proposed acid-based standardized operation procedures for MP analysis (including HNO_3_, H_2_SO_4_, HF or acid mixes)^43–45^, these reagents have recently been shown to cause MP alteration, damage, or even the loss of particles (Table 2, Table S3,^33,46,47^). Similarly, HCl digestions were recognized as destructive and insufficient^12,14,25^, while oxidizing reagents such as H_2_O_2_ or peroxodisulfate potassium (K_2_S_2_O_8_) are known to obscure particle detection, to leave particulate residues, to clog filters, or to lead to particle loss due to foaming(^24,48,49^, this study (Table S2)). Previous studies suggested a minimum concentration of 30% H_2_O_2_ for the complete digestion of tissue, although the reports about this are not consistent^40,50^. However, 30% H_2_O_2_ degraded PA, PET and Rayon and this can lead to a total loss of the polymer, while PE, PES, PP, PS particles were visually altered by this reagent (Table 2, Table S3,^14,25,50, 51^).

**Table 2:**
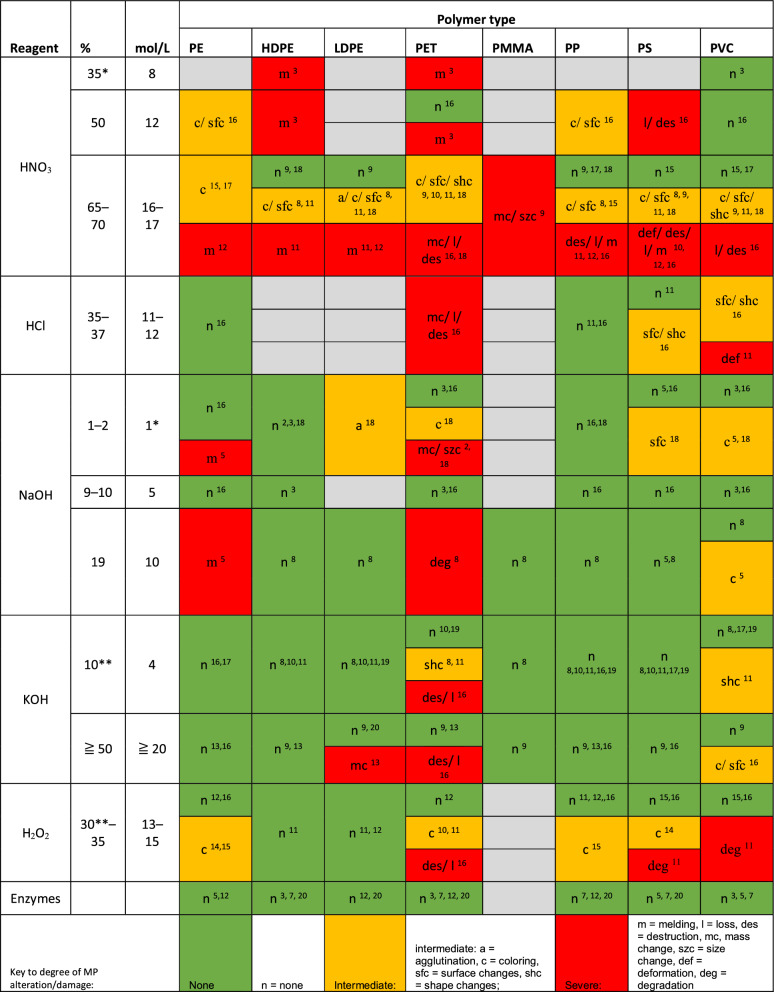
Literature review on common reagents in digestion protocols and their known effects on MP particles.

Key to MP Types: HDPE: high density polyethylene; LDPE: low density polyethylene; PE: polyethylene; PET: polyethylene terephthalate; PMMA: poly(methyl-methacrylate); PP: polypropylene; PS: polystyrene; PVC: polyvinyl chloride.

Sources: 1. Avio et al. 2016, 2. Budimir et al.^41^, 3. Catarino et al.^33^, 4. Claessens et al.^43^, 5. Cole et al.^12^, 6. Collard et al. 2015, 7. Courtene-Jones et al.^34^, 8. Dehaut et al. 2017, 9. Enders et al.^46^, 10. Hara et al.^42^, 11. Karami et al.^25^, 12. Karlsson et al.^47^, 13. Kühn et al.^35^, 14. Li et al.^51^, 15. Li et al.^50^, 16. Lusher et al.^14^, 17. Phuong et al.^45^, 18. Roch and Brinker 2017, 19. Thiele et al.^37^, 20. von Friesen et al.^38^.

*Minimum amount needed for complete digestion; **miminum amount needed lower than in table: H_2_O_2_ 15% (Tsangaris et al. 2021); KOH 1 M (Lavers et al.^56^).

More detailed table with more MP types, reagent types and reagent concentrations in Table S3.

Hence, we decided to use KOH (10%) as a base for all our protocols, because it is known as an efficient and benign digestion reagent (Table1, Table S1,^24,45^). Similar to the other reagents, it can damage certain polymer types, but this is only the case when concentrations exceed 10% (Table 2, Table S3). At a concentration of 10%, which is sufficient for the complete digestion of organic materials, KOH is known to affect cellulose acetate (CA), PC, PET and Rayon particles^24,37,42^, but its destructive impact can be reduced by keeping the digestion temperature ≤ 40 °C^37,42^. Following this reasoning and our literature review results, we choose a low concentrated KOH solution and milder temperatures < 40 °C and incubations for 1–3 days as a starting point for developing suitable protocols for zooplankton. We initially tested a 1-step KOH digestion with the above-mentioned zooplankton groups, but it only worked well for fish larvae. To make the protocol applicable also to chaetognaths, copepods and euphausiids, we included digestive enzymes.

For this kind of enzymes, such as trypsin, collagenase, proteinase K, no detrimental effects on MP have been shown so far^12,33, 34, 38,47^. Simultaneously, they allow a good digestion efficacy, with the only drawback that their use can be time- and cost-expensive^12,33,34,38^.

**Figure 1:**
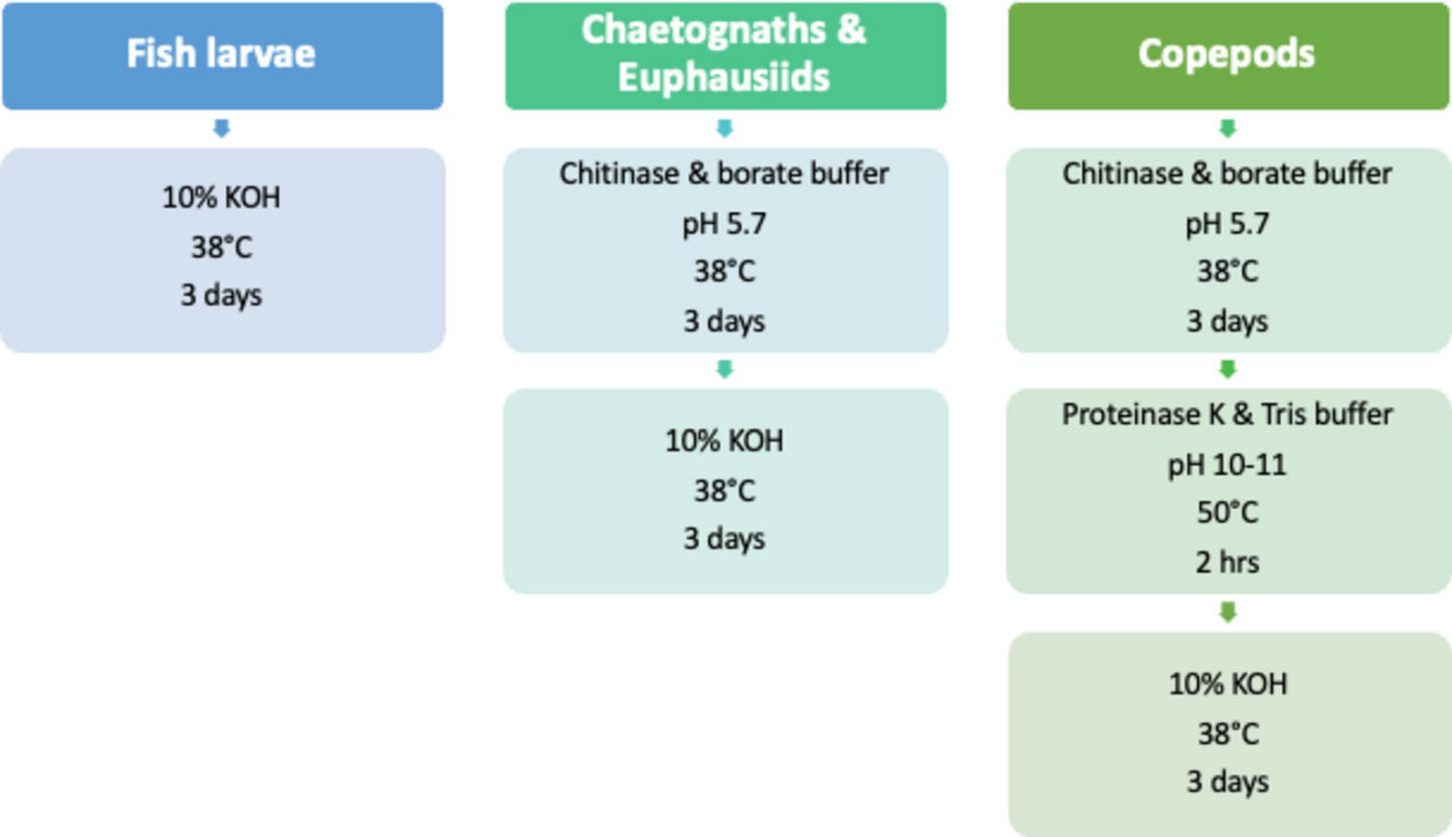
Digestion protocols for the four zooplankton taxa. The different digestion steps, pH values, the used reagents, incubation temperature, and incubation duration are indicated.

Finally, we identified three customised protocols of which two combine KOH with enzymes: For fish larvae, we used a 1-step digestion with 10% KOH at 38 °C for three days as most appropriate, while for chaetognaths and euphausiids a 2-step protocol was implemented. In this, an incubation with chitinase and borate buffer (pH 5.7) at 38 °C for three days, which served to break up carapaces and spines, was followed by a digestion with 10% KOH (same settings as above). Copepods were digested in three steps, which comprised the use of chitinase (same settings as above), followed by proteinase K with Tris buffer (pH 10–11) at 50 °C for 2 h and, again, a 3-day incubation in 10% KOH (same settings as above) (Fig. 1).

**Figure 2:**
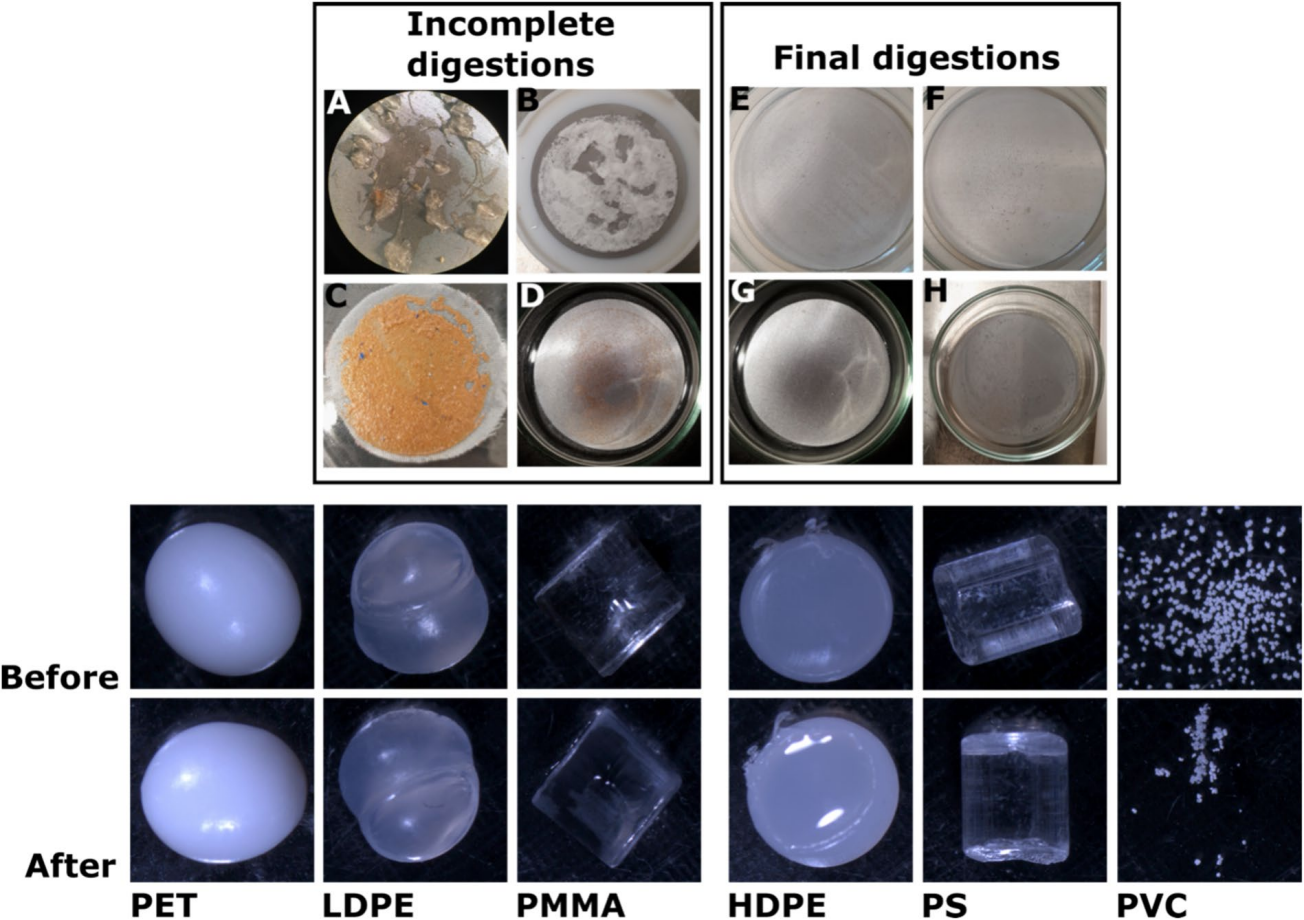
Digestion efficacy of unsuccessful (**A**–**D**) and successful (**E**—chaetognaths, **F**—euphausiids, **G**—fish larvae, **H**—copepods) protocols. Below are examples of different MP polymer types before and after digestion. The polymers remained mostly unaltered.

The air and wet blanks we had during the handling of the samples detected no contaminations. We assessed the quality of our protocols in terms of digestion efficacy, MP recovery rate and potential MP alteration. For all four zooplankton taxa, the digestion efficacy based on changes in filter weight of the protocol was > 98% (Table 3), while the visual inspections of the filters confirmed that no discoloration occurred and that only a few carapace pieces or spines remained on the filters (Fig. 2a-d). The latter did not impair the picking of particles (Fig. 2e-h).

**Table 3:** Digestion efficacy, based on changes in filter weight, of the protocols for the different zooplankton groups.

Zooplankton group	Digestion protocol	Digestion efficacy (%)	SD	n
Fish larvae	KOH (10%)	99.56	0.76	3
Chaetognaths	Chitinase & KOH (10%)	99.98	0.03	3
Euphausiids	Chitinase & KOH (10%)	98.38	2.15	3
Copepods	Chitinase, Proteinase & KOH (10%)	98.15	0.86	3

Mean recovery rates (± SD) for fish larvae, chaetognaths and euphausiids as well as copepods were 93.57 ± 3.09%, 92.17 ± 7.13% and 91.82 ± 8.98%, respectively (Table 4).

**Table 4:** Influence of the digestion protocols on MP size (%), visual properties, spectra obtained with a hyperspectral imaging camera (HSI) and recovery rates (%).

Digestion protocol	Zooplankton group	Recovery rate				MP Alteration			
		Recovery rate (%)	SD	n	Polymer type	Δ size (%)	SD	Visual alteration	Changes in HSI spectrum
KOH (10%)	Fish larvae	93.57	3.09	3	HDPE	1.01	1.29	None	None
					LDPE	0.81	1.25	None	None
					PS	0.53	1.09	None	None
					PET	0.97	0.86	None	None
					PMMA	1.31	1.09	None	NA
					PVC	NA	NA	None	NA
Chitinase & KOH (10%)	Chaetognaths & Euphausiids	92.17	7.13	3	HDPE	0.28	0.42	None	None
					LDPE	2.35	1.39	None	None
					PS	0.09	0.53	None	None
					PET	0.64	0.34	None	None
					PMMA	2.17	4.71	None	NA
					PVC	NA	NA	None	NA
Chitinase, Proteinase & KOH (10%)	Copepods	91.82	8.98	3	HDPE	1.31	0.73	None	None
					LDPE	1.70	0.86	None	None
					PS	1.38	1.14	None	None
					PET	0.91	1.33	None	None
					PMMA	1.36	0.27	None	NA
					PVC	NA	NA	None	NA

Values are given as means with standard deviation (SD). Replicate number (n). No spectra were available for PVC and PMMA particles.

There were no visual changes in any of the particles (HDPE, LDPE, PET, PS, and PMMA) after any of the digestions, except for a slight clumping of the PVC particles as a consequence of all protocols (Fig. 2, Table 4). However, this was most likely due to an incomplete drying of the particles after the digestion, or because the application of the particles a led to an uneven spreading. It was presumably not caused by a change in polymer conformation, since the particles were not altered with regard to their shape or surface texture. Spectra obtained with a near-infrared hyperspectral imaging (HSI) camera before and after digestion were examined for PE (HDPE, LDPE), PS and PET, but no changes were observed (Table 4). Changes in particle size were assessed for all particles except for those made of PVC. For the fish larvae protocol, the digestion-induced change in MP size ranged from 0.53 to 1.31%, for the chaetognath and euphausiid protocol it was between 0.28 and 2.35% and for the copepod protocol between 0.91 and 1.70%, respectively (Table 4).

The proposed protocols are based on a lowly concentrated KOH, which is optionally combined with chitinase and proteinase, and represent an approach that is not only more benign, but also includes fewer work steps than other enzymatic digestions^47^. Specifically, we omitted process steps prior to the digestion such as the weighing or the mechanical break-down of samples (e.g. by grinding with pestle and mortar or extracting only the digestive system). By adding proteinase K it was possible to digest even taxa (e.g. copepods) with thick or hard carapace. As a consequence, for all of the protocols, the filters were almost free of residues and it was therefore easy to pick the MP. Moreover, all quality control parameters (digestion efficacy, recovery rate and MP alteration) yielded satisfactory results.

However, the quality assurance we did has three limitations: First, we only considered the possible effects of the protocols on MP in a size range between ~ 4–250 µm. Furthermore, we focussed mostly on compact shapes such as spheres, cylindrical and in the case of PVC amorphous and did not check for a potential influence on particles that come with a large surface:mass ratio such as fibres, films and fragments. Finally, we used virgin MP and did not consider how weathered MP would react to the protocols we developed. All of these properties could potentially determine the susceptibility of MP to the proposed handling. Particle size seems unlikely to be of large relevance to estimate the usefulness of these protocols and the used reagents, because we inspected the surface, i.e. the contact area between the reagent and the plastic, for signs of alteration in terms of appearance and HSI spectra. Since a change in particle size would only affect the surface area, but not the chemical properties of the surface, it seems unlikely that we would have obtained different results for MP alteration, if we would have used larger or smaller MP for the tests^52,53^. If there was weathering to some extent due to the chemical reagents, this could potentially involve a change in the chemical properties, however in this study the identification via HSI remained unaffected by the protocols. However, we cannot rule out a very small change that would be barely visible with the HSI spectra, but potentially detectable with FTIR, or Raman spectroscopy. Despite this, identification via HSI, apart from having a higher detection limit, offers minimal sample preparation times and automated measurement that are more suitable for large sample quantities^54,55^. Furthermore, since zooplankton organisms can ingest only small and compact particles, it suggests that the shape is also neglectable in the context of extracting MP from this kind of animals. However, the question whether the proposed protocols alter weathered MP of different types, shapes and sizes cannot be answered on the base of our data and would require further research.

While the time requirements, especially for the 3-step digestion of copepods (i.e. 6 days) may represent a drawback of the protocols, their digestion efficacy and the negligible effects of the reagents on the MP are important advantages. A further disadvantage of all enzyme-based digestion protocols are the associated costs, but when they are applied to zooplankton samples the processed volumes are usually small and this prevents enhanced costs. In our case, the price for the 1-step protocol was 0.12€/sample, for the 2-step protocol (using chitinase) 1.49 €/sample and for the 3-step protocol (using Proteinase K) 2.15 €/sample. In comparison to various extraction protocols reviewed by Lavers et al.^56^, ours are among the cheapest when considering the KOH-only protocols, while, when including enzymatic digestions, they are still inexpensive and in any case cheaper than protocols using HNO_3_ or other enzymatic protocols. Further improvements of the protocols could include a fine-tuning of the weight:volume ratio of the reagents in order to reduce costs, or a reduction of the incubation times to 24–48 h. In summary, considering the insufficient digestion and sometimes damaging effects of pure base or oxidizing reagent protocols, as well as the destructive effects and high costs of acidic protocols, our enzymatic approach is effective, benign and cost-effective with a small, but, as we think, acceptable trade-off of requiring long incubation times. The proposed protocols will hopefully facilitate research on the ingestion of small-sized MP by zooplankton organisms, which constitute an essential fraction of the oceanic ecosystem.

## Material and methods

### Literature review

We searched for studies reporting digestion protocols that allow to investigate MP ingested by marine animals in a non-systematic literature review. For this, we screened the scientific database Google Scholar using keywords such as microplastic, digestion/extraction protocol, invertebrates/marine animals/zooplankton until 31.07.2023. Furthermore, we hand-picked potentially relevant papers that were cited in the selected studies. The targeted information we extracted were about the digestion efficacy, about the nature of the MP used, about evaluation of MP alterations and about MP recovery rates. Our search was exclusively focussed on studies in which marine animals (free-living, non-parasitic metazoans) were chemically digested (as a whole or specific tissues) using acids, bases, oxidising agents, or enzymes in order to extract previously ingested MP. We excluded (1)all papers that did not use chemical digestion as a method to extract MP. This included many studies on the gut contents of fish, marine birds and mammals, which, only used visual techniques (i.e. picking) to extract the MP from the digestion residues, and other studies which exclusively employed density-separation techniques. We excluded (2) papers that only investigated the effects of the chemical digestion protocol on MP without applying it to tissues, (3) studies that examined bulk samples without specifying any of the included taxonomic groups, (4) studies on terrestrial animals or non-animal materials (i.e. plants, water samples, sediment), (5) studies in which the digestion procedure was not clearly stated or which did not include digestion protocols, and (6) studies that did not report own data.

### Prevention of contamination

Several measures were taken to prevent the contamination of the samples with MP during processing. They were only washed with filtered seawater (8 µm) or deionized water and were exclusively handled with metal and glass equipment. All reagents were filtered before using them and each work step that required exposing a sample to air was conducted under a clean bench (see details in Wesch et al.^57^). To assess the background contamination with MP during sample procession, a wet glass fibre filter (Grade 691, VWR) was laid open in a glass petri dish next to a set of samples (i.e. air blank), while in parallel to each set of samples, one sample that contained only reagents, was processed in the same way (i.e. wet blank).

### Sampling, pre-treatment and storage

**Figure 3:**
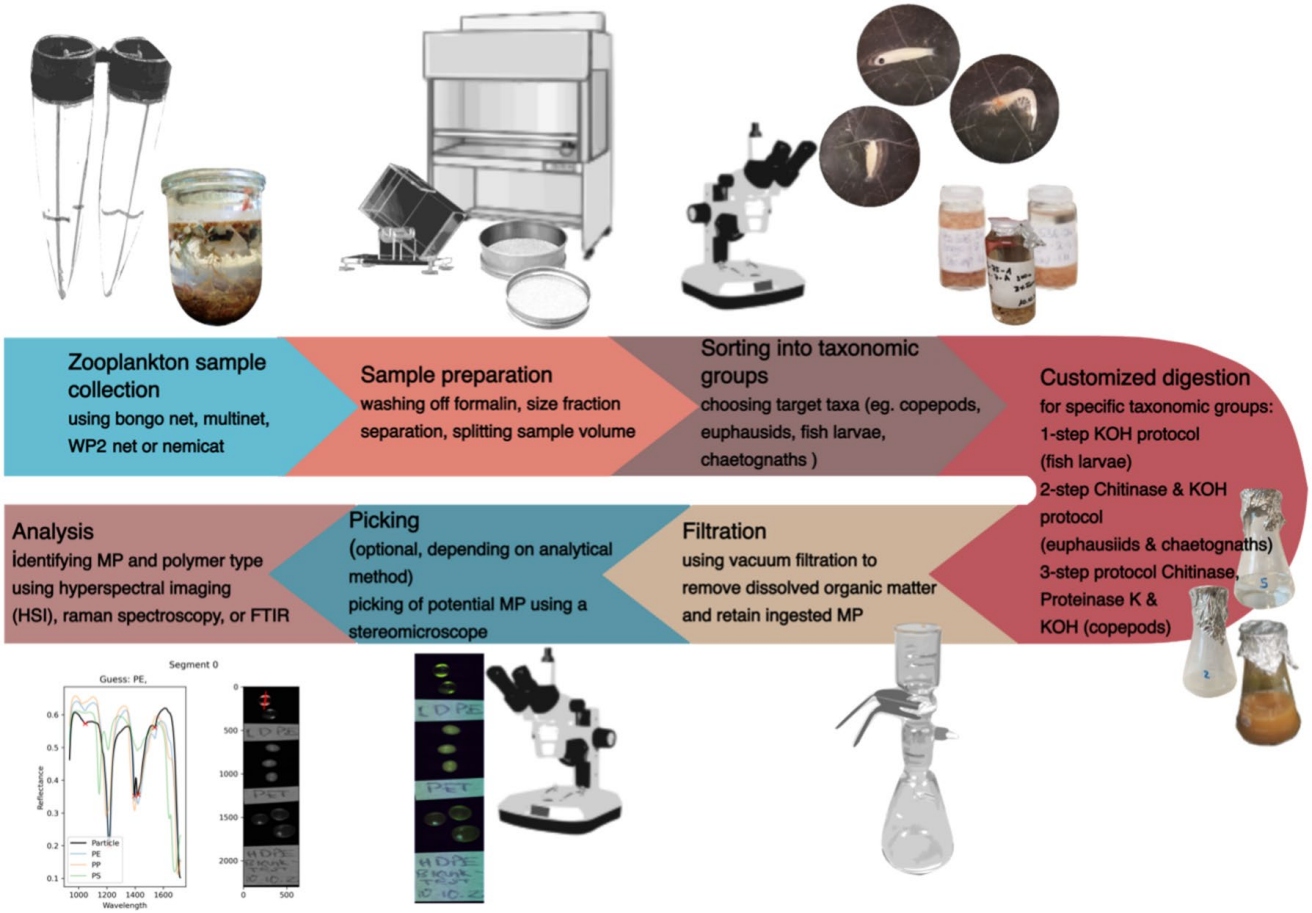
Zooplankton sample processing from collection to spectral analysis.

A Bongo net (Hydro-Bios, Kiel) equipped with a 300 μm mesh net was used to collect zooplankton organisms, which were rinsed into 500 ml glass jars and preserved in 5% filtered formaldehyde (Fig. 3). In the laboratory, samples were then split with a zoosplitter to reduce the biomass. MP that potentially adhered to the exoskeletons of the zooplankton organisms were washed off to ensure that only previously ingested MP were considered in the analysis (Fig. 3). To test this washing procedure trial samples were checked for adhering MP under a stereomicroscope before and after rinsing. Individuals belonging to the four above mentioned taxonomic groups were picked with forceps, transferred to glass vials and stored in 3% formaldehyde until digestion (Fig. 3).

### Trial protocols

To establish a tailored digestion protocol for each zooplankton group, trial protocols were adapted. The trial protocols consisted of combinations of different concentrations of chemicals in relation to biomass for the different organism groups (100 µl, 200 µl, 400 µl, 800 µl, 1 ml for Chitinase, 1–3 g for Protease, 100 µl and 200 µl for Lipase, 10% KOH, H2O2 + Fe(III) (Fenton’s, reagents)), differently applied orders of chemicals (first enzymatic digestion with KOH-digestion afterwards or first KOH-digestion with enzymatic digestion afterwards), different buffers (PBS buffer, citrate buffer, TRIS buffer, borate buffer, water), different temperature (room temperature, 30 °C, 37 °C, 40 °C) and digestion times (15 h, 24 h, 4–9 days).

### Digestion and filtration

Zooplankton samples were rinsed to remove the formaldehyde using a 62 µm metal sieve and distilled, filtered water. Afterwards, they were transferred into 100 mL Erlenmeyer beakers containing a magnetic stir bar made from glass. The beakers were covered with aluminium foil to avoid air contamination and evaporation. We did not grind the samples prior to the digestion to avoid an additional step that could have introduced contaminations. The volume of the reagent used was not adjusted to the individual samples, but was provided in amounts that were in any case sufficient for the digestion process. The wet weight of the samples ranged between 0.03 and 0.17 g for copepods, 0.2–0.35 g for fish larvae, 0.01–0.14 g for euphausiids and 0.01–0.17 g for chaetognaths. Furthermore, volumes of KOH and buffers were set to ensure the complete submersion of the organic material in the beakers, while simultaneously allowing thorough stirring. We tested a wide variety of established, adapted and new digestion procedures with fish larvae, chaetognaths, copepods and euphausiids until a successful digestion resulting in clear filters were achieved (Table S2). Fish larvae were digested using 50 mL 10% KOH solution, in which they were incubated at 38˚C for three days under continuous stirring (200 rpm). For euphausiids and chaetognaths, 10 mL of borate buffer (pH 5.7; 1 M boric acid, 250 mM NaOH) and 0.5–1 mL chitinase (> 100 U/mL) were added to the beaker and the samples were incubated at 38˚C for three days under continuous stirring (200 rpm). Afterwards, the residues were treated with KOH in the same way as described for the fish larvae. Since evaporation was minimal, the volume of the remaining slurry did not vary and, hence, the added KOH was applied in always the same concentration. However, to yield a final concentration of 10% KOH in the 2-step and 3-step protocols, KOH with a concentration of 15% was used. Copepods were first incubated with borate buffer and chitinase as described above (using 1 mL of chitinase). Following this, we added 15 mL of TRIS buffer (400 mM Tris, 60 mM EDTA, 105 mM NaCl, 1% SDS) and the pH was adjusted to 10–11 using NaOH. Then 25 µL of proteinase K (20 mg/mL) were added to each sample and heated to 50 °C for two hours while stirring (200 rpm). The final digestion step was the same as for the fish larvae. After the digestion was accomplished, samples were filtered onto 10 µm metal filters (FTEU, 48 mm diameter) using a vacuum pump (Fig. 3). Beakers and the filtration set up were thoroughly rinsed using both, distilled filtered water and ethanol. Metal filters were stored in glass petri dishes and dried for 24–72 h at 78 °C.

### Quality control

To assess the quality of the digestion protocols, we assessed (1) MP** r**ecovery, for which we spiked Erlenmeyer flasks with reference MP (n = 3 per protocol), (2) digestion efficacy with regard to the respective zooplankton group (n = 3 per protocol/taxa) and (3) alteration of the reference MP during digestion (n = 3 per protocol, number of particles per beaker ranged from n = 1–3 for HDPE, LDPE, PS, PET and PMMA, and many small, not counted particles for PVC).

To check whether a loss of particles occurred through (1) the use of chemical reagents, (2) the number of work steps or (3) the handling, we assessed or estimated the recovery rates of spiked particles^58,59^. They were determined by transferring a pre-counted amount of spherical MP particles (size 38 µm, Duke Scientific Corporation) into Erlenmeyer beaker and applying the digestion protocol. Afterwards, the recovered MP particles were counted.

In previous studies, digestion efficacy was assessed via changes in filter weight. However, this method does not indicate how well filters can be picked after digestion. This is because residues, the fragmentation of residues into smaller particles or the discoloration of particles would not affect filter weight, but can significantly hamper particle identification. To overcome this weakpoint, we propose to include a visual inspection of the filter when evaluating the digestion efficacy of a protocol. This serves to check whether discoloration occurred and to assess the number of residue particles that is present on the filter. To determine digestion efficacy, empty metal filters (10 µm, FTEU, 48 mm diameter) were weighed, then samples (n = 3) of each of the four different zooplankton groups were blotted dry carefully and their wet weight was recorded. Afterwards, samples were transferred to beakers and digested according to the respective protocol. Lastly, samples were filtered onto pre-weighed metal filters, dried in an oven (81 °C) for 24–72 h and then weighed again. Digestion efficacy was calculated as follows: Efficacy (%) = 100 − sample dry weight × 100/sample wet weight. Furthermore, the filters from each digestion protocol were visually inspected using a stereomicroscope (magnification 0.63–5×) for organic residues. We assessed their similarity to MP and their potential to obscure MP that are present on the filter.

MP alteration can be detected by a visual and spectroscopic/ HSI inspection of the particles before and after the digestion^46^. Here, MP alteration was assumed to have happened whenever a change in particle size, appearance (i.e. colour, shape, surface texture) and/or in the HSI spectrum^60^ occurred. We tested this for the newly developed protocols by exposing new MP particles (size range: 4–250 µm) that represented six different polymers (HDPE, LDPE, PET, PS, PMMA and PVC) to them. To test for MP alteration, three replicates of each of the six different polymers were exposed to each of the three protocols. Among the different polymers were three spherical particles: LDPE (mean diameter 186 ± 21 µm SD), HDPE (mean diameter 224 ± 16 µm) and PET (mean diameter 140 ± 7 µm), two cylindrical particles: PS (mean length 160 ± 7 µm) and PMMA (mean length 160 ± 16 µm), as well as one amorphous particle: PVC (7 ± 2 µm). Prior to digestion, photos were taken of the particles and their size was determined, while 1–3 particles per polymer were added to a beaker and then the digestion protocols were applied. Afterwards, the particles were photographed again and their size as well as any visual alteration were recorded. To check for potential changes in the HSI spectra that would impair particle identification, MP particles made from the same polymer that experienced digestion and such that did not experience digestion were both scanned (Fig. 3). We then compared the two groups to assess whether polymer identification outcome was impaired by the chemical digestion protocols. The HSI methodology to scan MP was recently described by Beck et al.^60^.

### Supplementary Information

Supplementary Table S1.

Supplementary Table S2.

Supplementary Table S3.

## Data Availability

All data generated or analysed during this study are included in this published article and its supplementary information files.
